# Keggin-Type Anions as Halogen Bond Acceptors

**DOI:** 10.1021/acs.cgd.2c01509

**Published:** 2023-03-24

**Authors:** Luka Fotović, Nikola Bedeković, Vladimir Stilinović

**Affiliations:** Department of Chemistry, Faculty of Science, University of Zagreb, Horvatovac 102a, 10000 Zagreb, Croatia

## Abstract

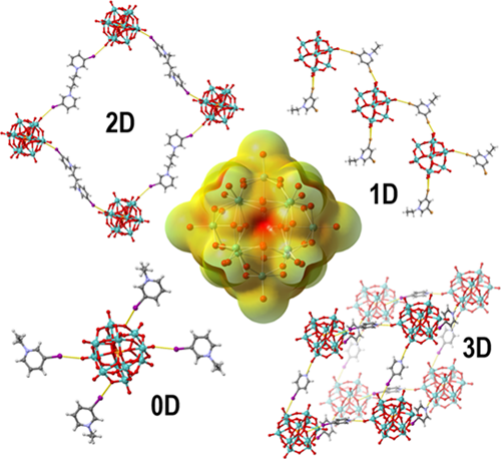

To study the potential of Keggin-type polyoxometalate anions to act as halogen bond acceptors, we have prepared a series of 10 halogen-bonded compounds starting from phosphomolybdic and phosphotungstic acid and halogenopyridinium cations as halogen (and hydrogen) bond donors. In all the structures, the cations and the anions were interconnected by halogen bonds, more often with terminal M=O oxygen atoms than with bridging oxygen atoms as acceptors. In four structures comprising protonated iodopyridinium cations capable of forming both hydrogen and halogen bonds with the anion, the halogen bond with the anion is apparently favored, whereas hydrogen bonds preferentially involve other acceptors present in the structure. In three obtained structures derived from phosphomolybdic acid, the corresponding oxoanion has been found in its reduced state [Mo_12_PO_40_]^4–^, which has also led to a decrease in halogen bond lengths as compared to the fully oxidated [Mo_12_PO_40_]^3–^. The electrostatic potential on the three types of anions involved in the study ([Mo_12_PO_40_]^3–^, [Mo_12_PO_40_]^4–^, and [W_12_PO_40_]^3–^) has been calculated for optimized geometries of the anions, and it has been shown that the terminal M=O oxygen atoms are the least negative sites of the anions, indicating that they act as halogen bond acceptors primarily due to their steric availability.

## Abstract 2

The potential of Keggin-type oxometalates for halogen-bond-based crystal engineering has been investigated through a series of 10 halogen-bonded crystalline materials that have been prepared based on phosphomolybdate and phosphotungstate anions and various halogenopyridinium cations as potential halogen (and hydrogen) bond donors.

## Introduction

Although hydrogen bond has remained the “interaction of choice” for the design of supramolecular structures, the research into designing multicomponent systems in which halogen bond^1^ acts as the principal intermolecular interaction has been intensifying over the past two decades.^2−8^ This is in part due to the strength and directionality of halogen bonds, which often exceed those of hydrogen bonds^9−11^ and enable the halogen bond to be used as a predictable and robust “binding agent” for planed preparation of desired supramolecular architectures.^12−16^ However, the majority of studies of using halogen bond in supramolecular chemistry have been focused on systems comprising neutral molecules, leaving ionic halogen-bonded structures still somewhat underinvestigated. The majority of ionic halogen-bonded systems studied to date are systems with halogenide anions as acceptors. This includes two major groups of compounds: those with simple halogenides^17−21^ and those with halogenometalate^22−26^ (or pseudohalogenometalate)^27−29^ anions as halogen bond acceptors.

Unlike halogenides, the oxygen atoms of oxoanions have been considerably less investigated as halogen bond acceptors. The halogen bonding proclivities of oxometalate (OM) and polyoxometalate (POM) anions have particularly been investigated only in a handful of studies. This is rather surprising given the interest the polyoxometalates have generally drawn over the past five decades due to not only their structural versatility but also numerous (both potential and actual) applications in catalysis,^30−33^ molecular electronics,^34,35^ and medicine.^36−38^ One way of introducing halogen bonding functionality into a POM system that has been investigated is functionalization of the POM core with a covalently bonded halogenated substituent.^39−45^ An alternative approach includes using halogenated counterions as halogen bond donors.^46−48^ Counterions are generally protonated nitrogen bases (pyridine and aniline derivatives) that interact with POM anions by both hydrogen and halogen bonds. In some cases, hydrogen can be replaced by silver ions that coordinate both POM oxygen and the nitrogen of the (halogenated) base molecule, leading to a functionalized heteronuclear POM species.^49,50^ Alternatively, as demonstrated by Mizuno and co-workers, coordinated species involving halogens can also be used as a potential halogen bond donating *macrocations*, sometimes resulting in porous ionic materials.^47^ On the other hand, Han and co-workers have shown that the presence of a potentially halogen bond donating pyridine derivative in the reaction mixture during the synthesis of POMs can influence the type of POM anion that will form.^51^ The above-mentioned studies clearly indicate that POMs do have high potential as building blocks in halogen-bonded materials and that a more systematic study of the behavior of POM as halogen bond acceptors could be beneficial for the future design of halogen-bonded POM materials.

**Scheme 1:**
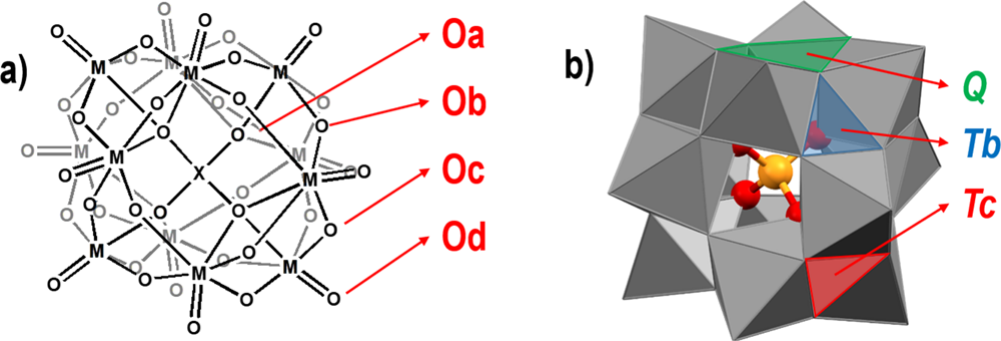
Keggin-Type Anion (a) Molecular diagram of the α-Keggin structure with labeled four types of oxygen atoms: Oa, central oxygen atom; Ob, bridging oxygen atoms connecting pairs of metal atoms not sharing a central atom; Oc, bridging oxygen atoms connecting pairs of metal atoms sharing a central atom; and Od, terminal oxygen atoms. (b) Polyhedral representation of the Keggin structure with labeled three types of faces: **Tb**, triangular face between three bridging oxygen atoms of the Ob type; **Tc**, triangular face between three bridging oxygen atoms of the Oc type; and **Q**, quadrangular faces (between two atoms of Ob and two of Oc type).

Therefore, in this paper, we provide a combined structural and computational study of a series of halogen-bonded solids in which POM anions bind to a series of structurally and electronically different halogen bond donating cations. As the model POM species, we have chosen the Keggin-type anion (Scheme 1). The Keggin anions were selected because of their general stability as well their appeal from a historic point of view: the first synthesized POM (ammonium phosphododecamolybdate synthesized by Berzelius in 1826)^52^ and the first to be characterized by X-ray diffraction (phosphododecatungstate by Keggin in 1933)^53−55^ were Keggin-type anions. The halogenated counterions used can be divided into three groups: (a) protonated iodopyridinium cations (which can form both halogen and hydrogen bonds to investigate the competition between those two interactions), (b) simple alkylated halogenopyridinium cations, and (c) bis(halopyridine) cations (which can bridge between two POMs forming two halogen bonds). To better understand the observed proclivity of the Keggin phosphododecamolybdate and phosphododecatungstate anions toward pyridines, theoretical studies of POMs observed in the crystal structures were also performed.

## Results and Discussion

**Figure 1:**
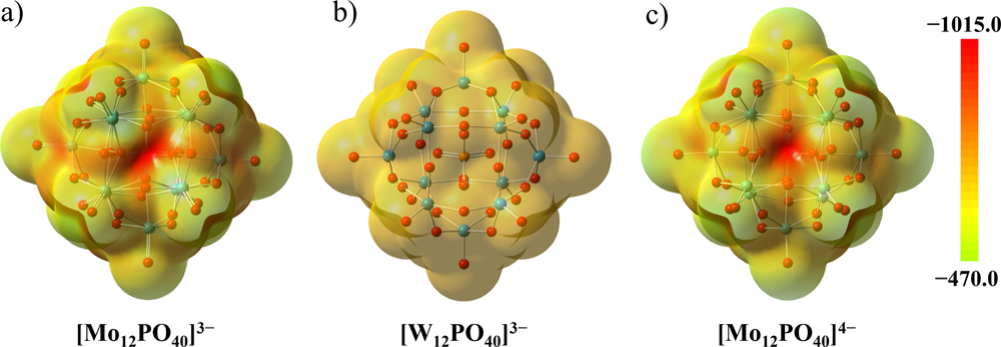
Molecular electrostatic potential mapped on the electron density isosurface (0.001 a.u.) of optimized (a) [Mo_12_PO_40_]^3–^, (b) [W_12_PO_40_]^3–^, and (c) [Mo_12_PO_40_]^4–^ viewed along a **Q** face.

To examine the potential of the Keggin-type anions for participation in halogen and hydrogen bonding, we performed DFT studies of the molecular electrostatic potential (MEP) of the [Mo_12_ PO_40_]^3–^, [Mo_12_PO_40_]^4–^, and [W_12_PO_40_]^3–^ anions *in vacuo* (Figure 1).

The α-Keggin structure (Scheme 1a) is of *T*_d_ symmetry, with four types of oxygen atoms independent by symmetry. The polyoxometalate anion can participate in halogen and hydrogen bonding through three types: the terminal oxygens (Od) and two types of bridging oxygen atoms (Ob and Oc), whereas the internal oxygen atoms (Oa) are within the anion and thus unavailable for intermolecular bonding. However, the MEP mapped on the electron density isosurface of the optimized geometries of the three anions that have been covered by this study (Figure 1) has shown that the spaces between the oxygen atoms—the faces of the Keggin cuboctahedron—also correspond to the minima of MEP and are therefore potentially good acceptor sites for either hydrogen or halogen bond. As a result of the symmetry of the anion, there are three types of faces: the quadrangular faces (**Q**) that are also faces bounded by four M–O–M bridges (two Ob and two Oc) and two types of triangular faces, each bounded by three M–O–M bridges (**Tb** by Ob atoms and **Tc** by Oc atoms) (Scheme 1b).

Molecular electrostatic potential values on oxygen atoms and faces of interest are listed in Table 1. It can be noticed that in all three anions, the most negative MEP values have been found in the center of **Q** faces, which are followed by MEP values in the center of triangular faces **Tc**. The average MEP values of bridging atoms Ob and Oc in [Mo_12_PO_40_]^3–^ and [W_12_PO_40_]^3–^ anions are very similar (differences are 3 and 5 kJ mol^–1^*e*^–1^, respectively), whereas in [Mo_12_PO_40_]^4–^, the difference is much greater, and Ob atoms are 17 kJ mol^–1^*e*^–1^ more negative than Oc. Triangular faces **Tb** are, on average, 20–38 kJ mol^–1^*e*^–1^ more positive than faces **Tc** in all studied anions, whereas the least negative MEP values have been found on terminal oxygen atoms Od.

**Table 1:** Average Values of the Molecular Electrostatic Potential on the Potential Acceptor Sites of the Studied Anions (Electrostatic Potential Values Are in kJ mol^–1^*e*^–1^)

acceptor site	[Mo_12_O_40_P]^3–^	[Mo_12_O_40_P]^4–^	[W_12_O_40_P]^3–^
atoms			
Oc	–703	–924	–674
Ob	–706	–941	–679
Od	–670	–899	–652
faces			
**Q**	–742	–1010	–720
**Tc**	–717	–954	–685
**Tb**	–679	–934	–661

In the first segment of our experimental study, we have focused on crystallizing phosphomolybdic acid with monoiodopyridines that were to act both as bases (for deprotonation of the acid) and as potential halogen bond donors. With all three iodopyridines, phosphomolybdic acid has yielded crystalline products (Table 2). However, they have greatly differed in solubilities and therefore in methods used for their preparation. The compound involving 2-iodopyridine **1** was highly soluble in the ethanol/water mixture used, and large single crystals were obtained by simple evaporation of the solvent at room temperature over several days. When a solution of 3-iodopyridine in ethanol was added to an aqueous solution of phosphomolybdic acid, a light-yellow precipitate was formed immediately, and it could be dissolved only by heating the mixture to the boiling point and increasing the amount of the solvent. Cooling the solution slowly to room temperature yielded crystals of **2**. A similar light-yellow precipitate appeared when 4-iodopyridine was used; however, this proved to be insoluble regardless of heating and addition of solvent. To obtain crystalline products, a layer of a diluted solution of 4-iodopyridine in ethyl acetate was carefully added over a diluted aqueous solution of phosphomolybdic acid. This procedure yielded two crystalline products: needles of a water/ethyl acetate solvate **3a** that appeared on the contact surface between the two solvents and rhombohedral crystals of the solvent-free **3b** that accumulated at the bottom of the aqueous layer.

**Table 2:** Compounds Prepared in This Study

compound	formula
**1**	(**2-Ipy**H)_3_(**2-Ipy**)[Mo_12_PO_40_]·3EtOH
**2**	(**3-Ipy**H)_3_(**3-Ipy**)_2_[Mo_12_PO_40_]·H_2_O
**3a**	(**4-Ipy**H)_3_[Mo_12_PO_40_]·EtOAc·H_2_O
**3b**	(**4-Ipy**H)_3_[Mo_12_PO_40_]
**4**	(**3-Ipy**Et)_4_[Mo_12_PO_40_]
**5**	(**3-Brpy**Et)_4_[Mo_12_PO_40_]
**6**	(**35-Brpy**Et)_3_[Mo_12_PO_40_]·4.5**dmso**
**7a**	[**(3IPy)_2_Bu**]_2_[Mo_12_PO_40_]Br
**7b**	[**(3IPy)_2_Bu**]_2_[Mo_12_PO_40_]·2**dmso**
**8**	[(**3-Ipy**Et)_2_(**3-Ipy**H)][W_12_PO_40_]·**dmso**

**Figure 2:**
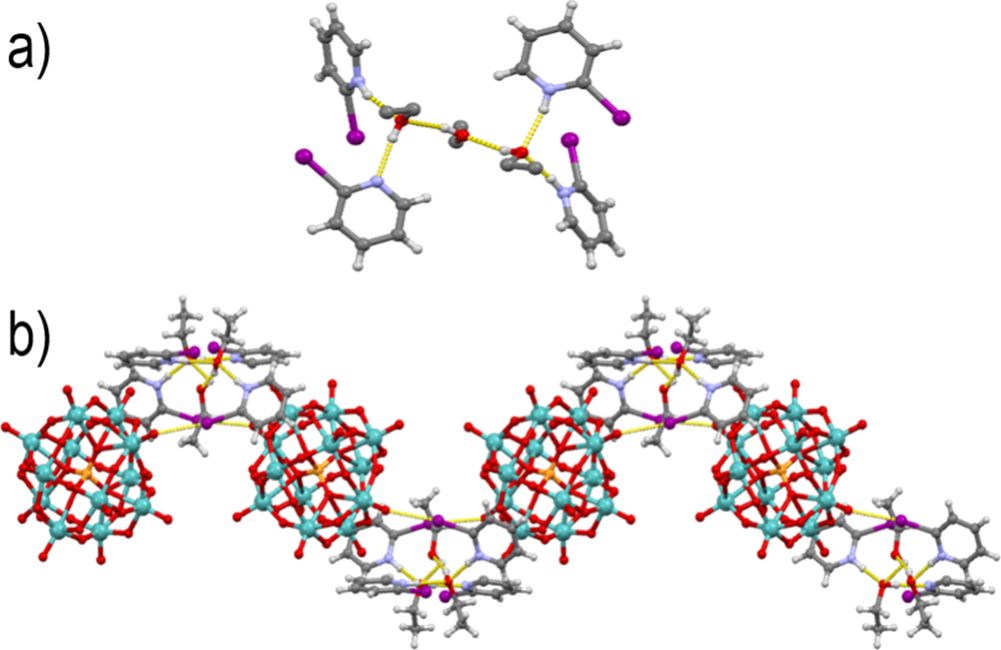
Intermolecular bonding in compound **1**: (a) hydrogen bonded [(**2-Ipy**H)_3_(**2-Ipy**)(EtOH)_3_]^3+^ cation (hydrogen atoms not involved in hydrogen bonding of the ethanol molecules have been omitted for clarity) and (b) hydrogen and halogen-bonded chain along the *c* axis viewed along [101].

Compound **1** crystallized in the noncentrosymmetric *Cc* space group. The three 2-iodopyridinium cations, one neutral 2-iodopyridine molecule, and three ethanol molecules form a hydrogen-bonded [(**2-Ipy**H)_3_(**2-Ipy**)(EtOH)_3_]^3+^ supramolecular cation (Figure 2a). All the O–H and N–H hydrogen bond donor groups present in the structure are thus involved in binding of the molecules comprising the [(**2-Ipy**H)_3_(**2-Ipy**)(EtOH)_3_]^3+^ cations. This leaves the iodine atoms free for forming halogen bonds with the anions. They do this in two ways: two 2-iodopyridinium cations form monocentric halogen bonds with terminal oxo ligands (I2···O31 of 3.136 Å and I3···O15 of 3.002 Å), whereas two 2-iodopyridine moieties form contacts with triangular faces of the anion: I1 with a Tc face (O29, O33, and O35) and I4 with a Td face (O16, O21, and O40), with the angle defined by the C–I bond of the donor and centroid of the three bridging oxygen atoms C16–I4···cent(3Ob) of 167° and C1–I1···cent(3Oc) of 170°) (Figure 2b). The more negative face of the Keggin anion is in contact with an iodine from a **2-Ipy**H^+^ cation, with the iodine being at ca. 3.06 Å from the centroid of the Oc atoms, whereas the less negative face is in contact with a neutral **2-Ipy** molecule (a weaker donor) and at a substantially longer distance (ca. 3.64 Å) from the centroid of the Ob atoms. The monocentric halogen bonds interconnect the [(**2-Ipy**H)_3_(**2-Ipy**)(EtOH)_3_]^3+^ cations and the anions into zig-zag chains along the crystallographic *c* axis, and the halogen bonds with the faces of the anions further interconnect the chains into a halogen-bonded 3D network.

**Figure 3:**
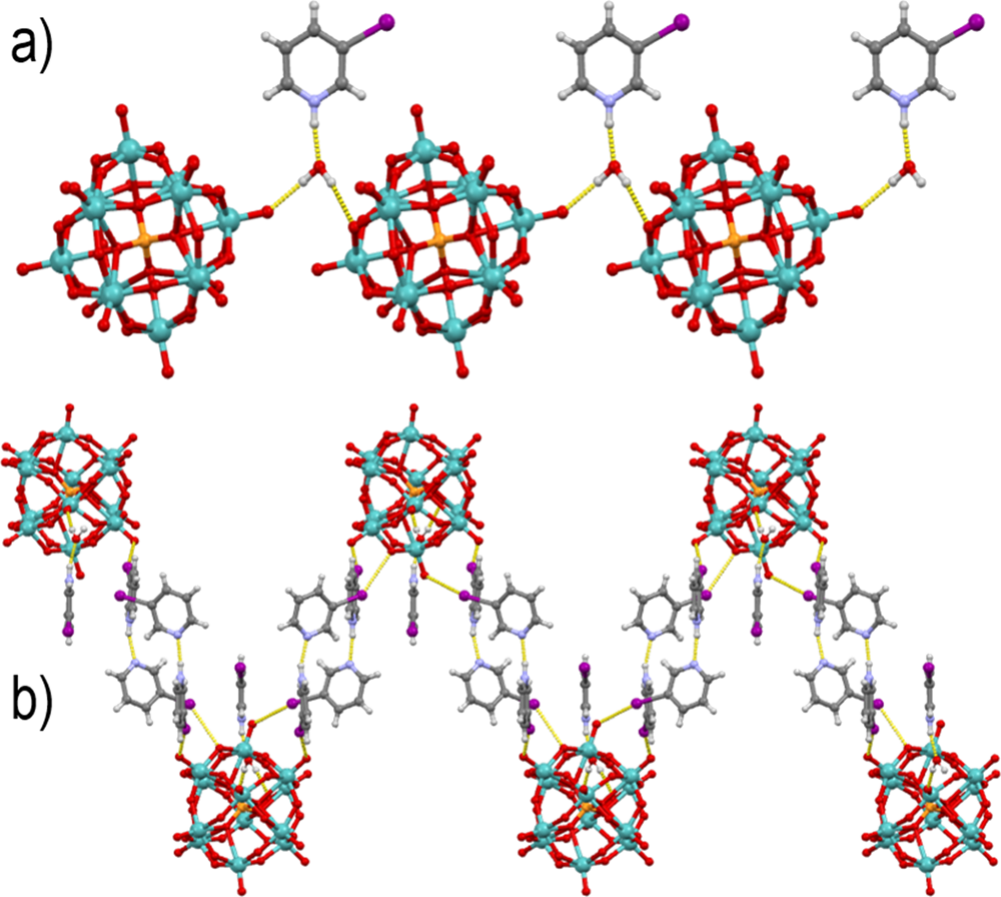
Intermolecular bonding in **2**: (a) hydrogen bonded chain along the *b* axis viewed along the *c* axis and (b) hydrogen and halogen-bonded layers perpendicular to the *a* axis viewed along the *b* axis.

3-Iodopyridine also yielded product **2** comprising neutral pyridine molecules. The two neutral **3-Ipy** molecules bind via (symmetric) N–H–N hydrogen bonds with two **3-Ipy**H^+^ to form supramolecular [(**3-Ipy**)_2_H]^+^ cations. The third **3-Ipy**H^+^ cation forms a N–H···O hydrogen bond with the solvent water molecule, which acts as a hydrogen bond donor to two [Mo_12_PO_40_]^3–^ anions interconnecting them into chains along the crystallographic *b* axis (Figure 3a). As in the case of **1**, all the iodopyridine iodine atoms form halogen bonds with the [Mo_12_PO_40_]^3–^ anions. The two [(**3-Ipy**)_2_H]^+^ cations interconnect the anions into corrugated layers perpendicular to the crystallographic *a* axis (Figure 3b). One [(**3-Ipy**)_2_H]^+^ forms a pair of closely linear halogen bonds with terminal oxo groups of two anions (I1···O1 of 3.243 Å, I5···O8 of 3.136 Å), whereas the other binds to one anion via a terminal M=O (I4···O12 of 3.326 Å) and is also in contact with a bridging oxygen (I2···O6 of 3.199 Å), albeit with an unusually low angle (138.3°), and the I2 iodine atom is also in (considerably longer) contact with a terminal M=O from a neighboring layer (I2···O36 of 3.442 Å). The layers are also interconnected via a series of bifurcated halogen bonds formed by the **3-Ipy**H^+^ cation hydrogen bonded to the water molecule with two (bridging) oxygen atoms of an anion from a neighboring layer (I3···O28 of 3.382 Å, 155.1° and I3···O35 of 3.262 Å, 159.4°) into a 3D structure.

**Figure 4:**
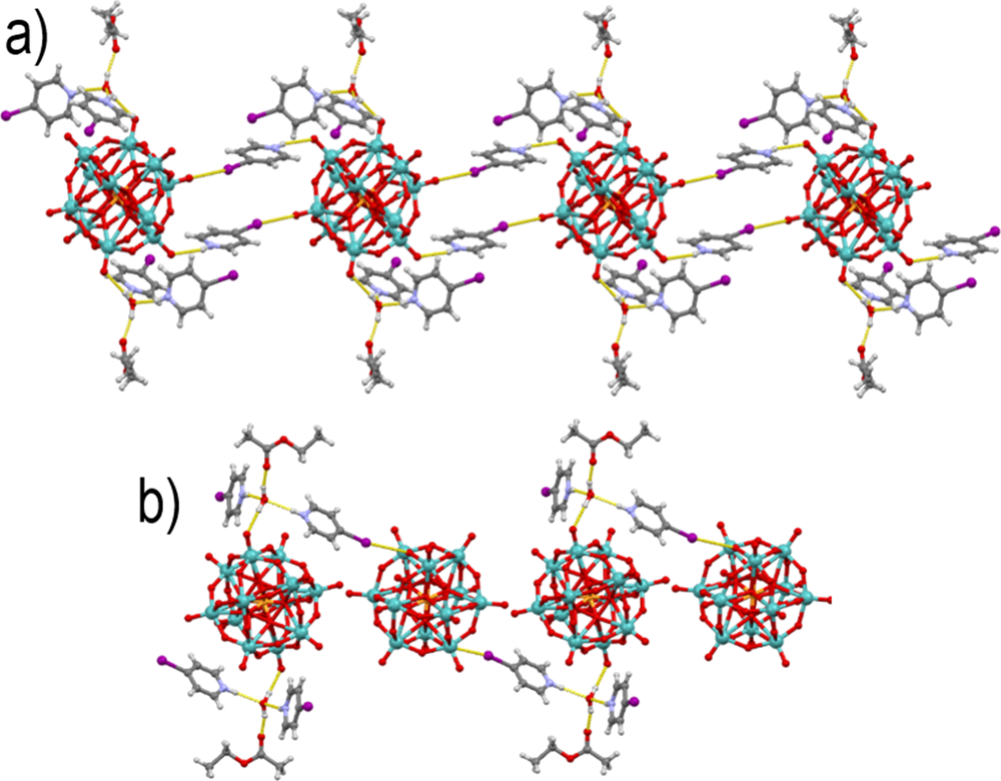
Crystal structure of **3a**: (a) chain of (symmetrically equivalent) anion cations along the crystallographic *c* axis and (b) chain of (symmetrically inequivalent) anions interconnected by [(**4-Ipy**H)_2_(H_2_O)(EtOAc)]^2+^ moieties along [111].

Unlike the crystal structures of the above two compounds, the two crystalline solids (**3a** and **3b**) obtained using 4-iodopyridine do not contain neutral iodopyridine molecules. In the crystal structure of **3a**, there are three **4-Ipy**H^+^ cations and two [Mo_12_PO_40_]^3–^ anions independent by symmetry (the latter positioned on inversion centers). Two **4-Ipy**H^+^ cations act as hydrogen bond donors to the water molecule, which is a hydrogen bond donor toward an ethyl acetate molecule (forming a [(**4-Ipy**H)_2_(H_2_O)(EtOAc)]^2+^ moiety), and to an [Mo_12_PO_40_]^3–^ anion. The remaining **4-Ipy**H^+^ cation binds directly to a [Mo_12_PO_40_]^3–^ anion as a hydrogen bond donor and to a neighboring anion as a halogen bond donor (I3···O8 of 3.496 Å) interconnecting the anions into chains along the crystallographic *c* axis (Figure 4a). Both hydrogen bonds are accepted by the same [Mo_12_PO_40_]^3–^ anion (of the two independent by symmetry) that thus acts as an acceptor of four hydrogen bonds (a pair of each type) and two halogen bonds from the bridging **4-Ipy**H^+^ cations, whereas the other [Mo_12_PO_40_]^3–^ anion participates only in halogen bonding with two [(**4-Ipy**H)_2_(H_2_O)(EtOAc)]^2+^ moieties. This results in chains with alternating hydrogen- and halogen-bonded [Mo_12_PO_40_]^3–^ anions along the crystallographic direction [111], with a [(**4-Ipy**H)_2_(H_2_O)(EtOAc)]^2+^ moiety bridging between each pair of neighboring anions (Figure 4b). Combination of this linking of the molecules with the **4-Ipy**H^+^ cation that bridge between anions along the *c* axis by hydrogen and halogen bonds leads to a 2D hydrogen- and halogen-bonded network perpendicular to [110]. This network is additionally stabilized by type II I···I (I2···I1 of 3.795 Å) contacts involving the remaining iodine atom from a [(**4-Ipy**H)_2_(H_2_O)(EtOAc)]^2+^ moiety as a halogen bond donor.

**Figure 5:**
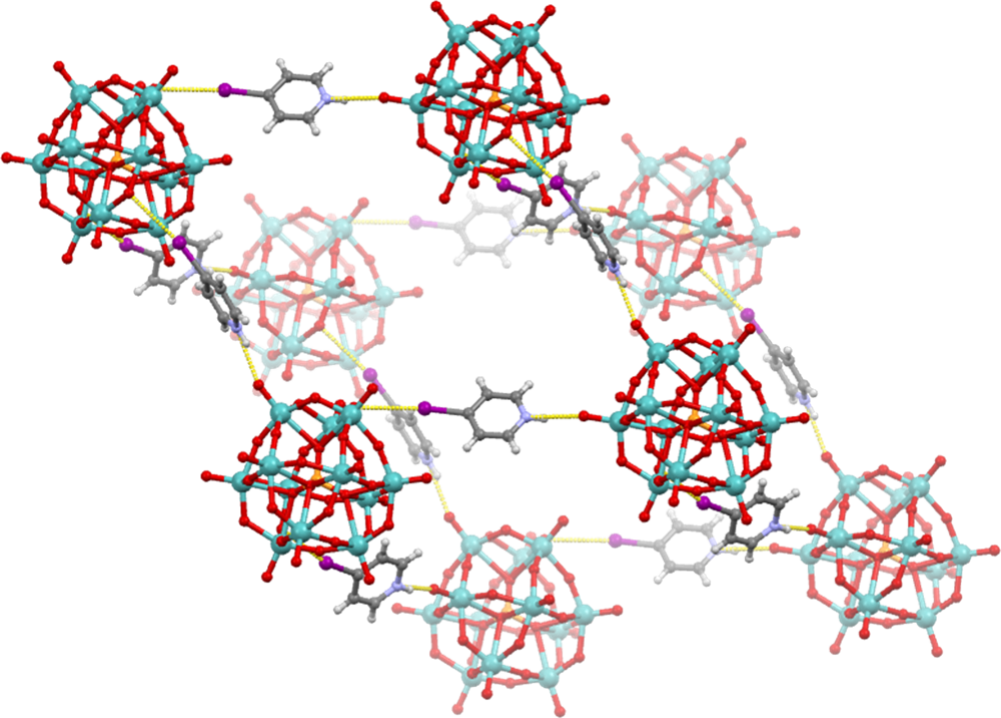
Fragment of the hydrogen and halogen-bonded 3D rhombohedral network in **3b**.

The solvent free **3b** crystallized in the trigonal *R*-3 space group. The [Mo_12_PO_40_]^3–^ anion formed three hydrogen and three halogen bonds (I1···O10 of 3.317 Å) with the **4-Ipy**H^+^ cations, each cation bridging between two anions (Figure 5). This resulted in a 3D elongated rhombohedral network, with anions at vertices and cations at sides of rhombohedra enclosing *ca.* 10 × 10 × 30 Å^3^. This space is filled by molecules belonging to three other rhombohedral networks so that the overall structure can be described as a quadruple interpenetrated network.

It can be observed that in all four structures, the [Mo_12_PO_40_]^3–^ anion acted as a halogen bond acceptor. Indeed, with multiple potential halogen bond donors present in the crystal structure, only in a single case is there a halogen bond donor not involved in a close contact with an oxygen atom from a [Mo_12_PO_40_]^3–^ anion (the one I···I contact in **3a**). On the other hand, the hydrogen bond donors often preferentially form hydrogen bonds with other acceptors in the structure (neutral pyridine or solvent molecules) rather than the [Mo_12_PO_40_]^3–^ anion. This seems to indicate that halogen bonding might be a more reliable intermolecular interaction for crystal engineering using polyoxometalate building blocks than the hydrogen bond. Therefore, we have attempted preparation of a further series of compounds derived from quaternary halogenopyridinium cations.

**Figure 6:**
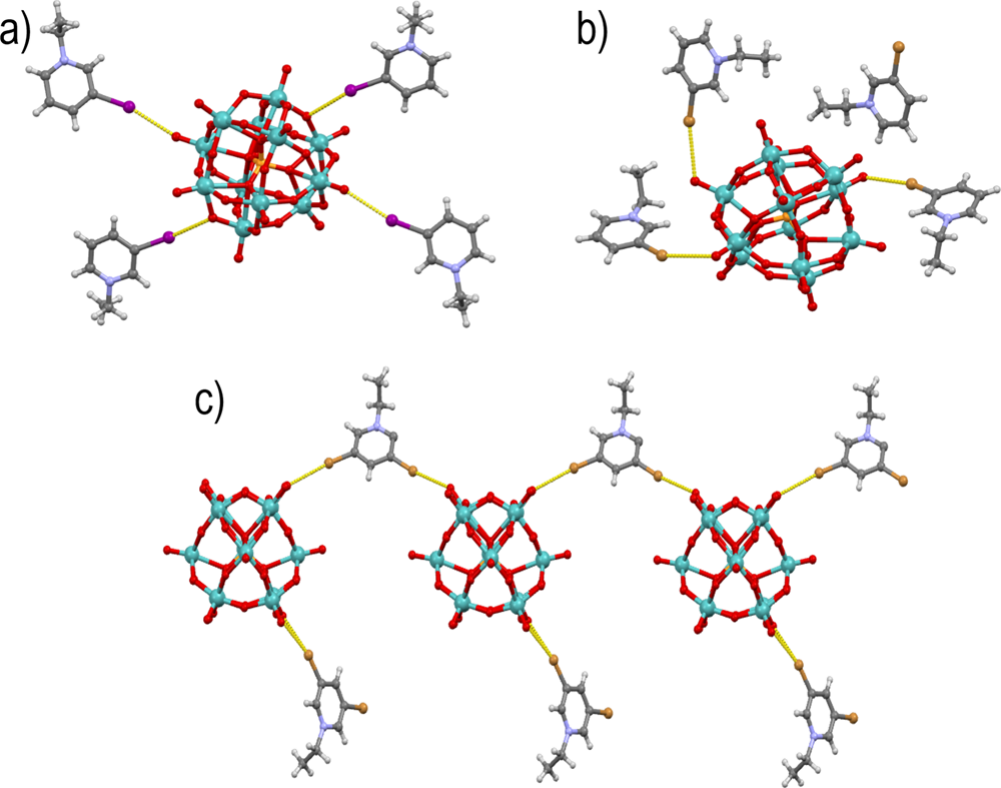
Asymmetric units and halogen bonding in (a) compound **4** and (b) compound **5** and (c) halogen-bonded chain in **6**.

Mixing aqueous and ethanol solutions of phosphomolybdic acid and *N*-ethyl-3-iodopyridinium iodide yielded an insoluble yellow precipitate. Subsequent investigation has revealed that the precipitate was somewhat soluble in hot dimethylsulfoxide (**dmso**), and this was used to attempt crystallization of the product. The **dmso** solution has yielded only several dark green (almost black) crystals of compound **4**, which were found to be *N*-ethyl-3-iodopyridinium salt of [Mo_12_PO_40_]^4–^ anion), i.e., that reduction of [Mo_12_PO_40_]^3–^ has occurred, most probably by **dmso**. The [Mo_12_PO_40_]^4–^ anion also formed halogen bonds with all the available donors, two via the terminal Mo=O groups (I1···O16 of 3.029 Å and I4···O27 of 3.055 Å) and two via bridging oxygen atoms (I3···O4 (Oc) of 2.986 Å and I2···O34 (Ob) of 3.098 Å) (Figure 6a).

The halogen bonds found in **4** are the shortest halogen bonds within the entire series of studied compounds. The considerable reduction of the I···O bond lengths in comparison to the above four cases can at least in part be attributed to the increase in the charge of the anion leading to more negative MEP values (Figure 1, Table 1). We have therefore decided to attempt expanding our survey onto bromine donors—*N*-ethyl-3-bromopyridinium (**3-Brpy**Et) and *N*-ethyl-3,5-dibromopyridinium (**3,5-Brpy**Et) cations—as the increased binding potential of the anion might compensate for the lower halogen bond donating power of the donors. Crystallization of the **3-Brpy**Et derivative has proceeded identically to that of the **3-Ipy**Et analogue, yielding dark green crystals of **5**. Unlike in the structure of the iodine analogue, here, only three out of four cations participated in halogen bonding, all three involving the terminal Mo=O groups (Br1···O1 of 3.233 Å, Br2···O28 of 2.944 Å, and Br3···O4 of 3.117 Å). The fourth cation was connected to three [Mo_12_PO_40_]^4–^ anions as a trifurcated C–H···O hydrogen bonding contact, whereas its bromine atom did not participate in any close contacts and is over 3.5 Å from the nearest nonhydrogen neighbor (Figure 6b).

The product **6** obtained from the hot **dmso** solution when *N*-ethyl-3,5-dibromopyridinium cations were used, rather surprisingly, did not include the reduced [Mo_12_PO_40_]^4–^ but rather a light yellow solid comprising the starting [Mo_12_PO_40_]^3–^ anions. Of the three **35-Brpy**Et^+^ cations, one formed two monocentric halogen bonds with terminal oxo groups of two anions (Br2···O22 of 3.185 Å and Br1···O39 of 3.130 Å) linking the anions into chains along the [−110] direction. Each anion further binds a cation through an asymmetric bifurcated halogen bond (Br6···O24 of 3.316 Å, 167.5° and Br6···O36 of 3.236 Å, 131.0°). The second bromine atom of this cation is in close contact with two Mo=O oxygen atoms from two anions belonging to two neighboring chains (Figure 6c). However, as both contacts occur at very sharp C–Br···O angles (130.9 and 86.3°), these do not seem to be interactions of the bromine σ-hole with the anions but rather incidental close contacts between oppositely charged ions. The third cation also forms only a sharp-angle contact with an oxygen atom from the anion (111.7°) but does participate in a linear halogen bond with a **dmso** molecule (Br4···O42 of 3.006 Å) employing the other bromine atom.

**Figure 7:**
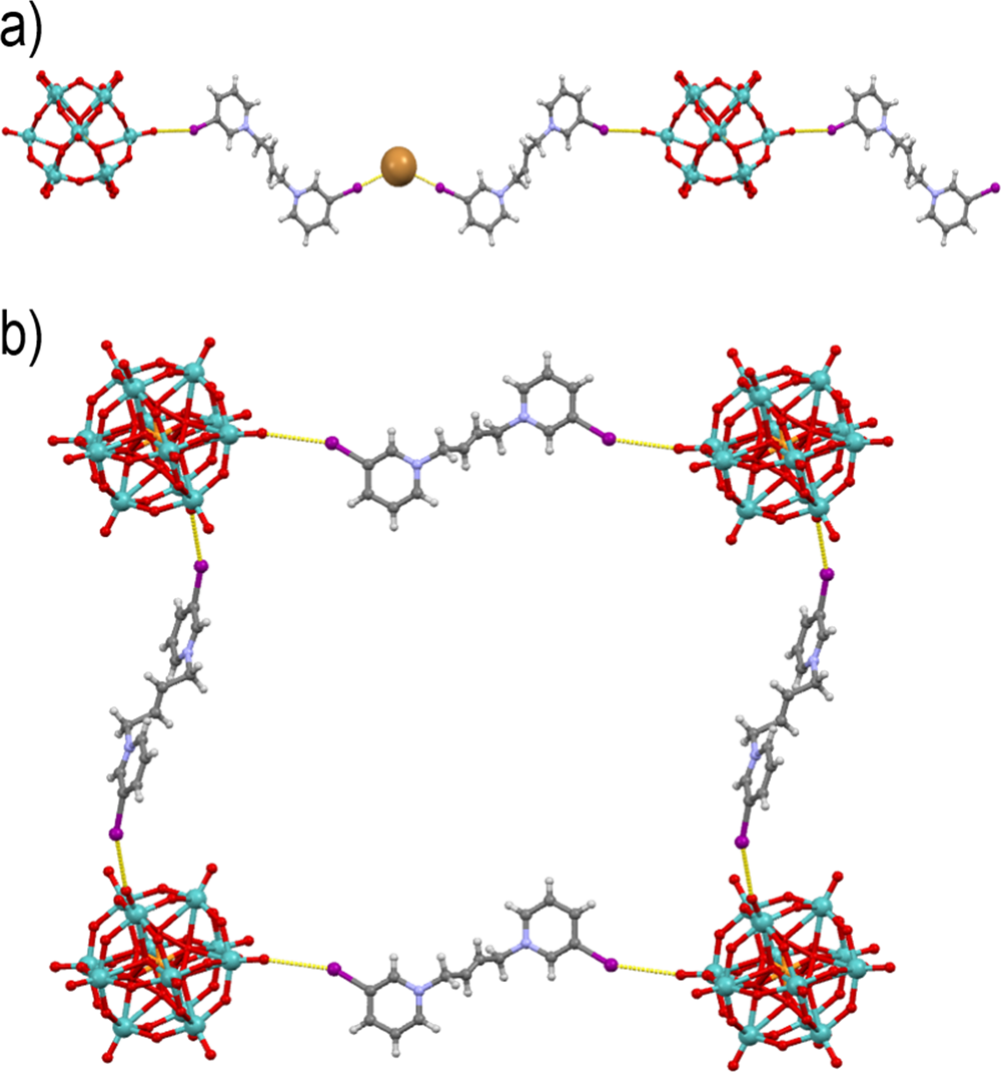
(a) Halogen-bonded chain in **7a** viewed approximately in the [101] direction. (b) Halogen-bonded 2-D network in **7b** viewed approximately in the [101] direction.

In addition to the simple *N*-ethylated halogenopyridinium cations, we have also attempted using a dication—(2*E*)-1,4-bis(3-iodopyridin-1-ium-1-yl)but-2-en ([**(3IPy)_2_Bu**]^2+^)—as a large, potentially bridging, ditopic halogen bond donor. Crystallization from a solution of [**(3IPy)_2_Bu**]Br_2_ and H_3_[Mo_12_PO_40_] in **dmso** yielded two crystalline products: the light green mixed salt **7a** and dark green **7b**. In **7a**, each cation formed two halogen bonds: one with an Od oxygen atom from a [Mo_12_PO_40_]^3–^ anion (I2···O19 of 3.296 Å) and one with a bromide anion (I1···Br1 of 3.213 Å) (Figure 7a). As each anion was an acceptor of two symmetrically equivalent halogen bonds, this resulted in halogen-bonded chains extending along the [−102] direction. In **7b**, the anion was once again the reduced [Mo_12_PO_40_]^4–^. There are two dications independent by symmetry. Both cations bridge between anions interconnecting them by halogen bonds (I1···O10 of 3.114 Å and I2···O6 of 3.279 Å) in two directions forming a 2D halogen-bonded network (Figure 7b).

**Figure 8:**
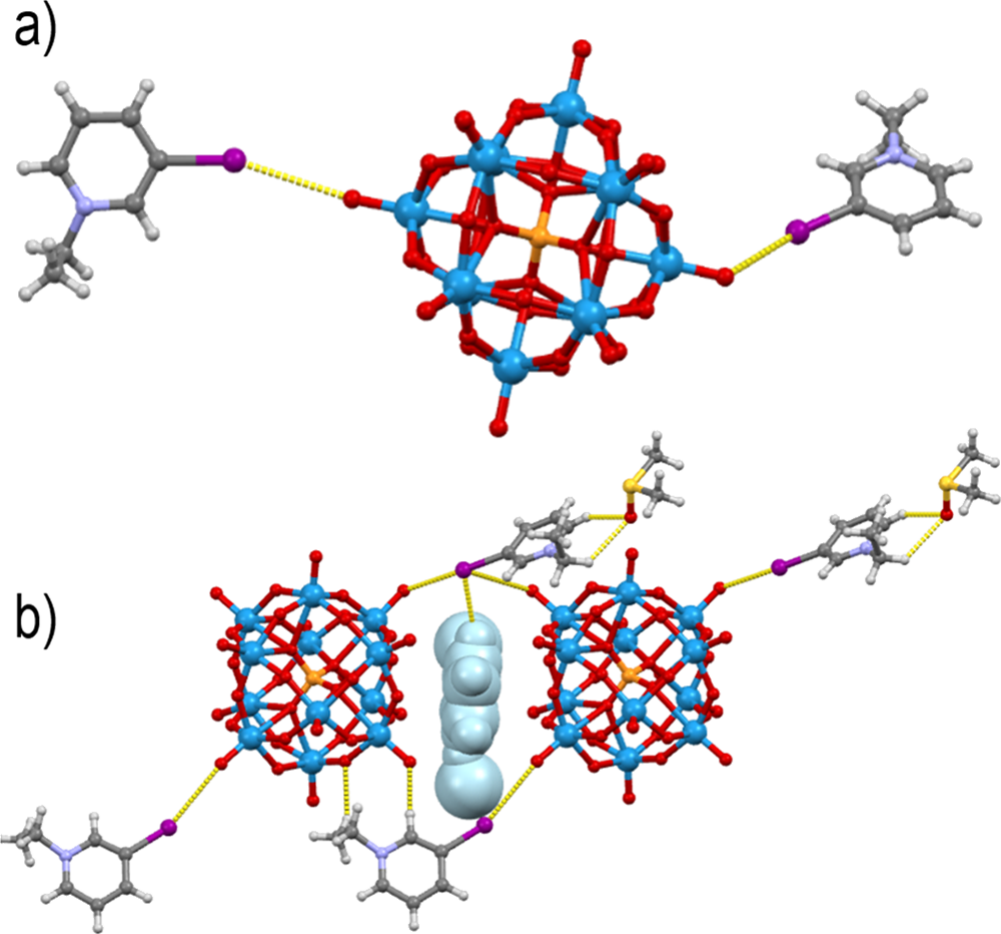
(a) Halogen-bonded (**3-Ipy**Et)_2_[W_12_PO_40_]^−^ unit in **8** and (b) a disordered **3-Ipy**H^+^ cation positioned between anions of two (**3-Ipy**Et)_2_[W_12_PO_40_]^−^·**dmso** units.

In addition to phosphomolybdates, we have also attempted to prepare equivalent phosphotungstates. They have however proven to be remarkably poorly soluble, and almost all attempts of synthesis have yielded amorphous precipitates. Only in the case of the product obtained from phosphotungstic acid and *N*-ethyl-3-iodopyridinium iodide did the crystallization from hot **dmso** yield crystalline product **8**. Unlike in the case of the molybdenum analogue, the [W_12_PO_40_]^3–^ anion was not reduced by the solvent, and the resulting solid was a 3:1 salt. Furthermore, the [W_12_PO_40_]^3–^ anion formed only two halogen bonds with the available **3-Ipy**Et^+^ cations (I1···O17 of 3.413 Å and I3···O33 of 3.038 Å), both via terminal Od atoms (Figure 8a). This formed discrete (**3-Ipy**Et)_2_[W_12_PO_40_]^−^ units that stack into layers parallel to the *ac* plane. The protonated **3-Ipy**H^+^ cation did not form halogen bonds; it was structurally disordered and, together with the solvent **dmso**, filled the spaces between the layers in the structure (Figure 8b).

The studied series of crystal structures show that there is a definite tendency of Keggin-type anions to form halogen bonds with halopyridinium cations (Table 3). Indeed, in structures where both hydrogen and halogen bond donors are present, the halogen bonds are favored. In the four structures with protonated iodopyridinium cations, the [Mo_12_PO_40_]^3–^ anions almost always form more halogen bonding than hydrogen bonding contacts (the exception being highly symmetric **3b** with three hydrogen and three halogen bonds per anion), even to the total exclusion of hydrogen bonds involving the [Mo_12_PO_40_]^3–^ anion in (**2-Ipy**H)_3_(**2-Ipy**)[Mo_12_PO_40_]·3EtOH.

**Table 3:** Lengths (*d*), Relative Lengths, and Angles (φ) of Halogen Bonds Established between Pyridinium Cations and Polyoxometalate Anions in Prepared Compounds

compound	contact	*d*/Å	φ/°	O atom type	relative length
**1**	C6–I2···O31	3.136(5)	167.8(2)	Od	0.896
	C11–I3···O15	3.002(3)	172.2(5)	Od	0.858
**2**	C2–I1···O1	3.243(4)	170.1(8)	Od	0.927
	C17–I4···O12	3.326(5)	167.9(3)	Od	0.950
	C12–I3···O28	3.382(7)	155.1(4)	Oc	0.967
	C12–I3···O35	3.262(5)	159.4(3)	Ob	0.932
	C22–I5···O8	3.136(2)	165.9(4)	Od	0.896
**3a**	C3–I1···O44	3.195(6)	168.5(6)	Oc	0.913
	C13–I3···O8	3.496(6)	171.8(6)	Od	0.999
**3b**	C3–I1···O10	3.317(8)	166.7(4)	Oc	0.948
**4**	C2–I1···O16	3.029(2)	167.3(2)	Od	0.865
	C9–I2···O34	3.098(2)	165.2(2)	Ob	0.885
	C16–I3···O4	2.986(2)	169.2(9)	Oc	0.853
	C23–I4···O27	3.055(1)	168.0(9)	Od	0.873
**5**	C2–Br1···O1	3.233(3)	164.7(8)	Od	0.959
	C9–Br2···O28	2.944(6)	163.1(9)	Od	0.874
	C16–Br3···O4	3.117(4)	149.5(9)	Od	0.925
**6**	C2–Br1···O39	3.130(4)	170.3(3)	Od	0.929
	C4–Br2···O22	3.185(3)	173.7(7)	Od	0.945
	C18–Br6···O24	3.316(2)	167.4(7)	Oc	0.984
**7a**	C11–I2···O19	3.296(5)	161.8(7)	Od	0.942
**7b**	C2–I1···O10	3.114(2)	157.6(2)	Od	0.890
	C9–I2···O6	3.279(9)	153.9(5)	Od	0.937
**8**	C2–I1···O17	3.413(5)	161.7(3)	Od	0.881
	C9–I3···O33	3.038(3)	168.8(9)	Od	0.972

As a result of the size of the anions, the charge is delocalized over a large number of oxygen atoms, which makes them less than ideal hydrogen bond acceptors, and consequently, hydrogen bonds form preferentially with other available acceptors. This reluctance of Keggin-type anions to form halogen bonds is also reflected in the crystallographic data deposited with the Cambridge Structural Database.^56^ Out of 724 data sets that involve a Keggin-type anion and a potential hydrogen bond donor (N–H or O–H), only in 161 (ca. 22%) does the anion act as a hydrogen bond acceptor. When a hydrogen bond is formed with a Keggin anion, the acceptor is most commonly (in 143 cases) a terminal oxygen atom in spite of the fact that the terminal oxygen is the least negative (as noted above; Figure 1). The probable reason is due to the fact that the terminal oxygen atoms protrude from the surface of the anion, making them most accessible for intermolecular contacts. Of the bridging oxygen atoms, the more negative Ob expectedly participate in more hydrogen bonds (in 118 structures) than the less negative Oc (83). Interestingly, in the structures covered by this study, all the hydrogen bonds have been formed with the terminal oxygen atoms (Od) as acceptors, whereas the halogen bonds roughly follow the pattern observed for hydrogen bonds in the CSD data; they most commonly form with the terminal oxygen atoms (18 contacts within the structures covered by this study) followed by the more negative bridging oxygen atoms (two contacts with Ob and 7 with Oc). In few cases, the halogen approaches the centroid of a triangular face (usually **Tc**), forming close contacts (albeit at unusually low angles) with multiple oxygen atoms. This indicates that the observed preference of the anions to form halogen bonds can partly also be attributed to steric reasons; as the halogen atoms are larger and “stick out” from the bulk of the donor molecule, they can penetrate more easily between the terminal oxygen atoms than the hydrogen atom and reach the more negative areas of the anion, leading to stronger interactions. The terminal atoms are still most easily reached, leading to a high number of contacts that involve them, but as the more negative inner atoms are more easily reached by the halogen than the hydrogen atoms, only halogen bonds are formed with the inner atoms. Also, as the halogen atoms exhibit larger positive surface areas to the anions, they can achieve better contacts with the negative *faces* of the anions. The effect of the difference of the electrostatic potential in the two inequivalent types of triangular faces (**Tc** and **Tb** atoms) is particularly well illustrated by the structure of **1** where there are two inequivalent triangular faces of the anion that exhibit both selectivity toward halogen bond donors (the more negative face in contact with the better donor) and marked difference in bond strength (the two differing in I···centroid distance by almost 0.6 Å).

## Conclusions

The Keggin-type polyoxometalate anions have demonstrated both considerable potential and versatility as halogen bond acceptors forming halogen-bonded discrete assemblies, chains, and 2D or 3D networks depending on the halopyridinium cation used. The distribution of the electrostatic potential on the anion favors the formation of multicentric bonds with faces of the anion, whereas sterically it is most favorable for a halogen to bind on a terminal M= O oxygen, and this apparently determines the observed distribution of halogen bond acceptor sites. However, halogen bond lengths generally do somewhat increase with the ESP of the contact oxygen atom, the longest bonds being with the [W_12_PO_40_]^3–^ phosphotungstate that has the lowest surface potential, whereas the shortest bonds are formed by the more highly charged [Mo_12_PO_40_]^4–^ anion. These observations indicate that Keggin ions (and probably polyoxometalates in general) can be used as building blocks in halogen-bonded solids in a predictable (and at least somewhat controllable) fashion. Furthermore, the possibility of preparing 2D (**7b**) and 3D networks (**3b**) with large separation between constituent ions indicates that this might be a feasible pathway for the systematic preparation of porous structures. This, in conjunction with the well-documented catalytic activity of polyoxometalates as well as low solubilities of such materials, leads to a tantalizing idea of potential applications of such materials in heterogeneous catalysis, which, in our opinion, merits further investigation.

## Experimental Section

### Synthesis

**1** and **2** were obtained by dissolving H_3_[Mo_12_PO_40_] (0.95 g; 0.5 mmol) and 2-iodopyridine for **1** (1.02 g; 2.5 mmol) or 3-iodopyridine (1.02 g; 2.5 mmol) for **2** in boiling ethanol (10 mL) to which water was added until all H_3_[Mo_12_PO_40_] dissolved. In the case of **1**, the resulting solution was left to cool and evaporate over several days, yielding yellow prismatic crystals of **1**. In the case of **2**, a light-yellow precipitate appeared that could be dissolved by increasing the amount of the solvent to ca. 30 mL (and somewhat increasing the water content in the solvent). Cooling the solution slowly to room temperature yielded crystals of **2**.

A similar procedure employing 3-iodopyridine also yielded a light-yellow precipitate, which proved to be insoluble regardless of heating and addition of solvent. To obtain crystalline products, a layer of a diluted solution of 4-iodopyridine (1.02 g; 2.5 mmol) in ethyl acetate (5 mL) was carefully added over a diluted aqueous solution of H_3_[Mo_12_PO_40_] (0.95 g; 0.5 mmol in ca. 5 mL of water). This procedure yielded two crystalline products: needles of **3a** that appeared on the contact surface between the two solvents and rhombohedral crystals of **3b** that accumulated at the bottom of the aqueous layer.

**4**–**6** were obtained by mixing aqueous solutions of H_3_[Mo_12_PO_40_] (0.95 g; 0.5 mmol in ca. 5 mL of water) and the corresponding *N*-ethylhalogenopyridinium iodide (2.5 mmol in 5–10 mL of water), dissolving the resulting pale green precipitate in boiling dimethyl sulfoxide, and leaving the solution to slowly cool to room temperature. **8** was obtained in an identical manner, only using H_3_[W_12_PO_40_] (1.44 g; 0.5 mmol).

Crystals of **7a** and **7b** were obtained in the same synthesis by dissolving H_3_[Mo_12_PO_40_] (0.95 g; 0.5 mmol in ca. 5 mL of water) and [**(3IPy)_2_Bu**]Br_2_ in dimethyl sulfoxide, heating the solutions to the boiling point, mixing the hot solutions in a Petri dish, and leaving the solution to slowly evaporate over 2–3 weeks.

The *N*-ethylhalogenopyridinium iodides and [**(3IPy)_2_Bu**]Br_2_ were prepared as described in the literature.^57^

### X-ray Diffraction Measurements

Single-crystal X-ray diffraction experiments for **1**, **2**, **3a**, **3b**, **6**, **7a**, and **7b** were performed using an Oxford Diffraction Xcalibur Kappa CCD X-ray diffractometer with graphite-monochromated Mo Kα (λ = 0.71073 Å) radiation, whereas diffraction measurements for the **4**, **5**, and **8** crystals were measured on the Oxford Diffraction XtaLAB Synergy, Dualflex, HyPix X-ray four-circle diffractometer with mirror-monochromated Mo Kα (*λ* = 0.71073 Å) radiation. The data sets were collected using the ω-scan mode over the 2θ range up to 60°. The CrysAlis PRO CCD and CrysAlis PRO RED programs were employed for data collection, cell refinement, and data reduction.^58,59^ The structures were solved by intrinsic phasing using SHELXT^60^ or by direct methods using the SHELXS and refined using SHELXL programs.^61^ The structural refinement was performed on *F*^2^ using all data. The hydrogen atoms were placed in calculated positions and treated as riding on their parent atoms. In the structure of **6**, a portion of the solvent **dmso** was severely disordered and could not be reasonably modeled but was excluded using Squeeze^62^ that found ca. 145 electrons in a solvent accessible void of ca. 280 Å^3^, both corresponding to three additional molecules of **dmso** per unit cell. All calculations were performed using the WinGX^63^ or Olex2 1.3-ac4^64^ crystallographic suite of programs. A summary of data pertinent to X-ray crystallographic experiments is provided in Table S1 (see ESI). Further details are available from the Cambridge Crystallographic Centre (CCDC 2223019–2223027 and 2226970 contain crystallographic data for this paper). Molecular structures of compounds and their packing diagrams were prepared using Mercury.^65^

## Computational Details

All calculations were performed using the Gaussian 16 software package.^66^ Geometry optimizations of cation molecules for analysis of the molecular electrostatic potential were performed using the b3lyp/def2tzvp level of theory starting from geometries obtained by X-ray crystallography.^67^ Harmonic frequency calculations were performed on the optimized geometries to ensure the success of each geometry optimization. The figures were prepared using GaussView.^68^

## Supporting Information Available

Crystallographic data for all compounds and ORTEP representations of the formula units of the prepared compounds (PDF)
