# Supplementary material for: Keggin-Type Anions as Halogen Bond Acceptors
Source: Cryst Growth Des. 2023 Mar 24;23(5):3384–92. doi: 10.1021/acs.cgd.2c01509 (PMC10162449; doi:10.1021/acs.cgd.2c01509)
Supplement: Supplementary file 1 — cg2c01509_si_001.pdf [file cg2c01509_si_001.pdf]

SUPPORTING INFORMATION

Keggin-type Anions as Halogen Bond Acceptors

Luka Fotović, Nikola Bedeković and Vladimir Stilinović

Department of Chemistry, Faculty of Science, University of Zagreb, Horvatovac 102a, HR-10000 Zagreb, Croatia

Email: vstilinovic@chem.pmf.hr
Fax: +385 1 4606 341
Tel: +385 1 4606 371

Table of Contents

Table of Content

Item	Page
**Table S1** (Crystallographic data)	2
**Figures S1–S10** (ORTEP representations of the formula units of the prepared compounds)	5

Table S1. Crystal data and refinement details for the prepared cocrystals.

	**1**	**2**	**3a**
CCDC No.	2223022	2223023	2223024
Molecular formula	Mo_{12} O_{40} P, 3(C_5H_5IN),C_5H_4IN, 3(C_2H_6O)	C_5H_5IN, 4($C_5H_{4.5}IN$), $Mo_{12}O_{40}P$, H_2O	3(C_7H_9IN), $Mo_{12}O_{40}P$, ($C_4H_8O_2$), H_2O
M_r	2783.45	2868.25	2546.37
Crystal system	monoclinic	orthorhombic	triclinic
Space group	*Cc*	$Pna2_1$	*P*–1
Crystal data:			
a / Å	28.966(6)	25.208(2)	11.5580(6)
b / Å	11.2183(8)	12.2028(6)	12.9633(5)
c / Å	25.937(10)	20.2308(10)	19.4160(11)
α / °	90	90	85.310(4)
β / °	131.89(3)	90	77.143(5)
γ / °	90	90	71.208(4)
V / Å^3	6274.2	6223.1	2684.82
Z, Z'	4, 2	4, 1	2, 1
D_{calc} / g cm^{-3}	2.947	3.061	3.150
λ(MoK_α) / Å	0.71073	0.71073	0.71073
T / K	293	293	293
Crystal size / mm^3	0.04 x 0.07 x 0.05	0.07 x 0.05 x 0.02	0.07 x 0.04 x 0.01
μ / mm^{-1}	4.407	4.936	4.566
F(000)	5184	5288	2364
Refl. collected/unique	30835 / 9275	54867 / 5815	38929 / 7057
Parameters	823	751	724
$\Delta\rho_{max}$, $\Delta\rho_{min}$ / e Å^{-3}	1.116; –0.950	1.905; –1.544	2.358; –2.840
$R[F^2 > 4\sigma(F^2)]$	0.0360	0.0503	0.0827
w$R(F^2)$	0.0817	0.1006	0.1712
Goodness-of-fit, *S*	1.042	0.864	1.172

Table S1. Continued.

	**3b**	**4**	**5**
CCDC No.	2223020	2223026	2223021
Molecular formula	$3(C_5H_5IN)Mo_{12}PO_{40}$	$4(C_7H_9IN)$, $Mo_{12}O_{40}P$	$Mo_{12}O_{40}P$, $4(C_7H_9BrN)$
M_r	270.10	2758.46	2570.46
Crystal system	trigonal	monoclinic	triclinic
Space group	*R*–3	*Pn*	*P*1
Crystal data:			
a / Å	19.6504(3)	10.9898(2)	11.1789(3)
b / Å	19.6504(3)	14.0388(2)	12.0091(4)
c / Å	20.6861(3)	19.5003(3)	12.8669(4)
α / °	90	90	109.290(3)
β / °	90	95.8890(10)	104.738(3)
γ / °	120	90	105.221(3)
V / Å^3	6917.55	2992.7	1458.67
Z, Z'	6, 1	2, 1	1, 1
D_{calc} / g cm^{-3}	3.515	3.061	2.926
$\lambda(MoK_\alpha)$ / Å	0.71073	0.71073	0.71073
T / K	293	293	293
Crystal size / mm^3	0.05 x 0.02 x 0.01	0.08 x 0.03 x 0.02	0.07 x 0.04 x 0.03
μ / mm^{-1}	5.303	4.615	5.356
F(000)	6744	2566	1211
Refl. collected/unique	29033 / 3629	84859 / 12453	19727 / 7942
Parameters	224	807	807
$\Delta\rho_{max}$, $\Delta\rho_{min}$ / e Å^{-3}	0.750; – 0.696	1.628; –1.161	1.849; –2.037
$R[F^2 > 4\sigma(F^2)]$	0.0171	0.0322	0.0677
$wR(F^2)$	0.0392	0.0774	0.1955
Goodness-of-fit, *S*	1.108	1.051	1.102

Table S1. Continued.

	**6**	**7a**	**7b**
CCDC No.	2223019	2223025	2226970
Molecular formula	$Mo_{12}O_{40}P$, 2($C_{14}H_{14}I_2N_2$, 4.5(C_2H_6OS)	$Mo_{12}O_{40}P$, 2($C_{14}H_{14}I_2N_2$), Br	3($C_7H_8NBr_2$), $Mo_{12}O_{40}P$, 3(C_2H_6OS)
M_r	2906.65	2830.29	2855.53
Crystal system	triclinic	monoclinic	triclinic
Space group	P–1	C2/c	P–1
Crystal data:			
a / Å	11.3642(14)	16.9167(6)	13.0303(4)
b / Å	12.8983(12)	19.8513(6)	13.4016(12)
c / Å	13.7862(11)	18.9624(6)	21.3991(8)
α / °	76.205(7)	90	88.162(7)
β / °	67.001(10)	104.802(4)	84.490(9)
γ / °	65.781(11)	90	76.839(5)
V / Å^3	1689.05	6156.6	3621.7(3)
Z, Z'	1, 0.5	4, 1	2, 1
D_{calc} / g cm^{-3}	2.858	3.053	2.619
λ(MoK_α) / Å	0.71073	0.71073	0.71073
T / K	293	293	293
Crystal size / mm^3	0.06 x 0.04 x 0.03	0.12 x 0.09 x 0.02	0.09 x 0.06 x 0.05
μ / mm^{-1}	4.158	5.136	5.511
F(000)	1359	5240	2694
Refl. collected/unique	15836 / 4211	2075 / 1080	12577 / 7083
Parameters	463	407	859
$\Delta\rho_{max}$, $\Delta\rho_{min}$ / e Å^{-3}	3.147; –1.885	1.788; –1.579	3.222; –1.786
$R[F^2 > 4\sigma(F^2)]$	0.0645	0.0473	0.0655
w$R(F^2)$	0.1671	0.1132	0.1815
Goodness-of-fit, S	0.803	1.052	1.023

Table S1. Continued.

	**8**
CCDC No.	2223027
Molecular formula	$O_{40}PW_{12}$, 2(C_7H_9IN), C_2H_6OS, C_5H_5IN
M_r	3629.28
Crystal system	triclinic
Space group	*P*–1
Crystal data:	
a / Å	11.9305(2)
b / Å	11.9683(2)
c / Å	23.3217(4)
α / °	78.1490(10)
β / °	78.8000(10)
γ / °	63.522(2)
V / Å^3	2897.11
Z, Z'	2, 1
D_{calc} / g cm^{-3}	4.160
λ(MoK_α) / Å	0.71073
T / K	293
Crystal size / mm^3	0.06 x 0.05 x 0.01
μ / mm^{-1}	25.472
F(000)	3164
Refl. collected/unique	42591 / 10039
Parameters	765
$\Delta\rho_{max}$, $\Delta\rho_{min}$ / e Å^{-3}	4.161; –2.595
$R[F^2 > 4\sigma(F^2)]$	0.0648
w$R(F^2)$	0.1691
Goodness-of-fit, S	1.107

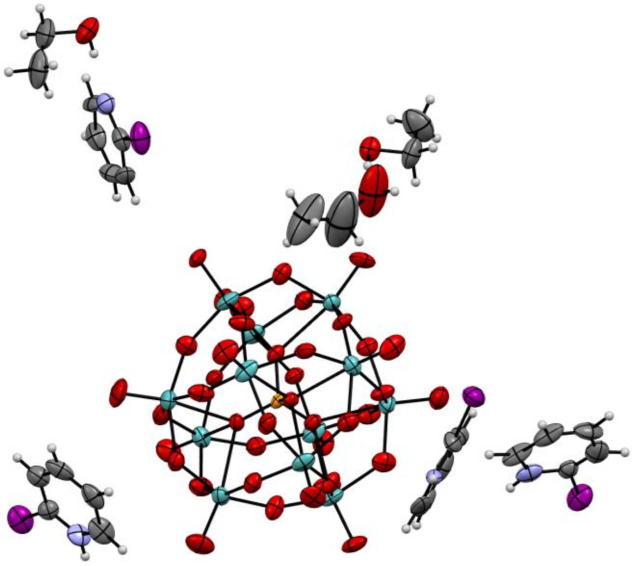

Figure S1. Molecular structure of **1** showing the atom-labelling scheme. Displacement ellipsoids are drawn at the 50 % probability level, and H atoms are shown as small spheres of arbitrary radius.

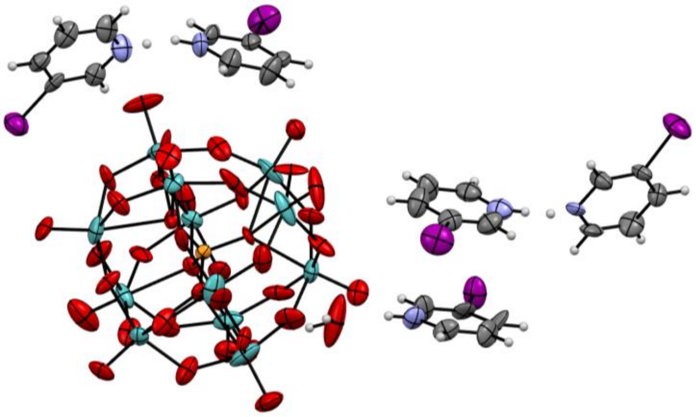

Figure S2. Molecular structure of **2** showing the atom-labelling scheme. Displacement ellipsoids are drawn at the 50 % probability level, and H atoms are shown as small spheres of arbitrary radius.

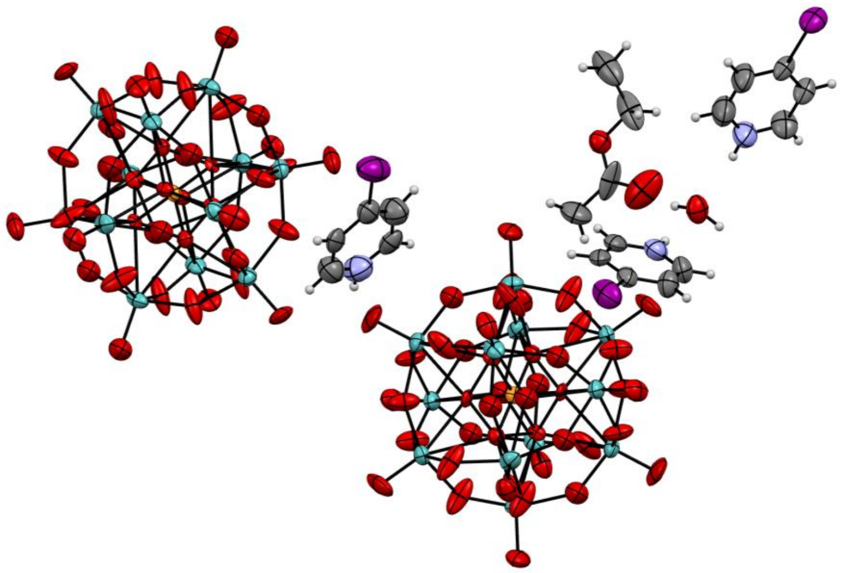

Figure S3. Molecular structure of **3a** showing the atom-labelling scheme. Displacement ellipsoids are drawn at the 50 % probability level, and H atoms are shown as small spheres of arbitrary radius.

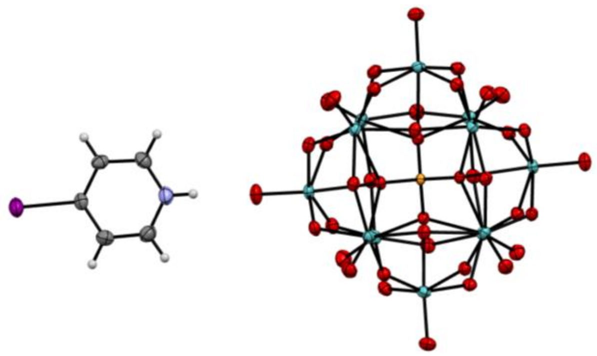

Figure S4. Molecular structure of **3b** showing the atom-labelling scheme. Displacement ellipsoids are drawn at the 50 % probability level, and H atoms are shown as small spheres of arbitrary radius.

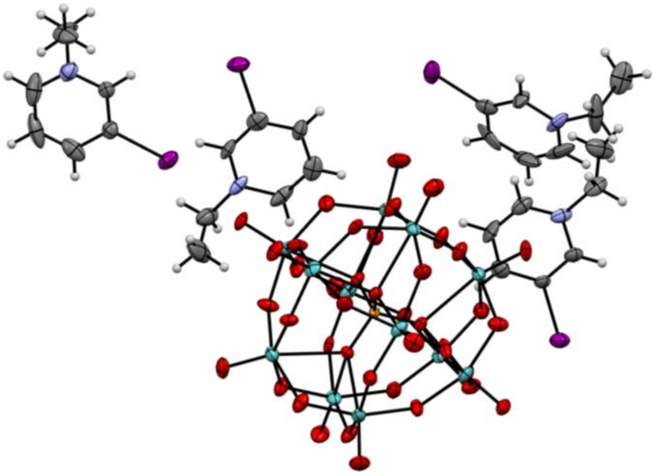

Figure S5. Molecular structure of **4** showing the atom-labelling scheme. Displacement ellipsoids are drawn at the 50 % probability level, and H atoms are shown as small spheres of arbitrary radius.

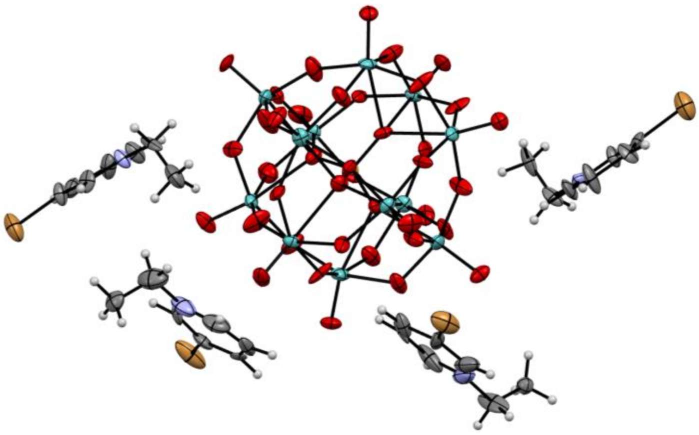

Figure S6. Molecular structure of **5** showing the atom-labelling scheme. Displacement ellipsoids are drawn at the 50 % probability level, and H atoms are shown as small spheres of arbitrary radius.

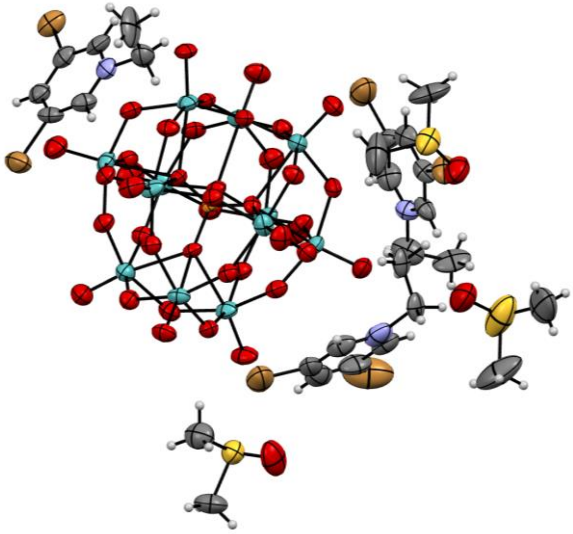

Figure S7. Molecular structure of **6** showing the atom-labelling scheme. Displacement ellipsoids are drawn at the 50 % probability level, and H atoms are shown as small spheres of arbitrary radius.

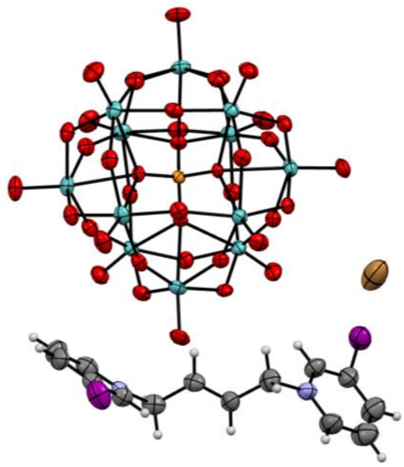

Figure S9. Molecular structure of **7a** showing the atom-labelling scheme. Displacement ellipsoids are drawn at the 50 % probability level, and H atoms are shown as small spheres of arbitrary radius.

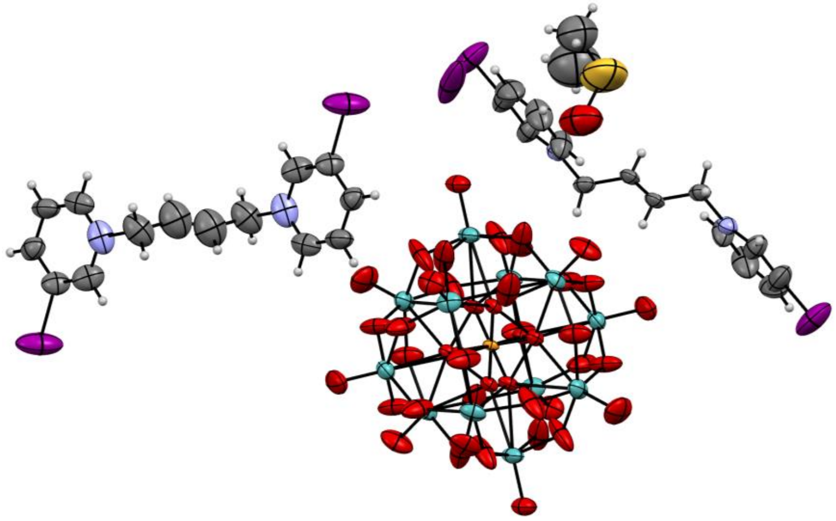

Figure S8. Molecular structure of **7b** showing the atom-labelling scheme. Displacement ellipsoids are drawn at the 50 % probability level, and H atoms are shown as small spheres of arbitrary radius.

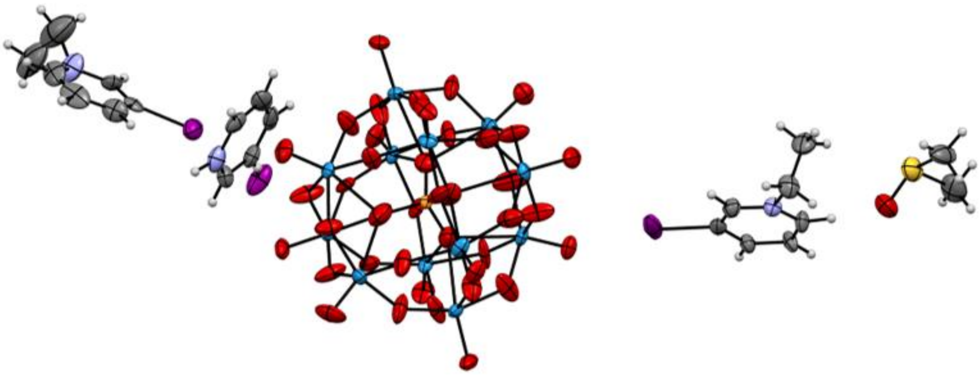

Figure S10. Molecular structure of **8** showing the atom-labelling scheme. Displacement ellipsoids are drawn at the 50 % probability level, and H atoms are shown as small spheres of arbitrary radius. Only the major component of the disordered **3-Ipy**Et^{+} anion is shown.

Several of the strucutres comprise an addtitional interesting feature – short O···O contacts between polyoxometalate anions. These cannot be due to hydrogen bonding (of partly protonated anions), since in all structures the number and charge of the present cations if exactly as required for compensating for the charge of the fully deprotonated anion. Also, there is no sign of additional electron density between any of the oxygen atoms in question which would indicate presence of an additional hydrogen atom, even in structures which could have been refined to a sufficiently high degree that such an maximum in residual electron map would have to be obvious (such as in $(N\text{-}Et\text{-}3Ipy)_4$ $Mo_{12}PO_{40}$ with R = 3.22 % or $(4IpyH)_3$ $Mo_{12}PO_{40}$ with R = 1.71 %)

It should be noted that short O···O contacts between polyoxometalate anions do occasionally occur in structures comprising Keggin anions. A quick search of the strucuttres comprising Keggin-type ions in the CSD (1004 strucutres overal) provides ca 150 hits with inter-anion O···O contacts, i.e. such contacts occur in ca 15% of structures comprising Keggin anions studied to date. These are predominantly contacts between the terminal atoms (97 hits, i.e. ca. 10 %), and are particularly common in structures comprising relatively small counter-ions, such as pyridinium cations (see e.g. AXORUB, COVCAU, BATCIQ, BATCOQ, HAJZEZ, POVXAB, RICCAJ), as well as other *N*-heterocycles. It appears therefore that inter-anion O···O contacts will appear whenever the number and the size of the cations (and other molecules in the sructure, such as solvent moleculas) , are insufficient to entirely 'insulate' an anion from the neghboring one, and still alow for closely packed structure. Indeed, in the light of the electrostatic ponential of the Keggin anions, the existance of such contacts is not particularly surprising – the electrostatic potential on the oxygen atoms (terminal M=O atoms in particular) is relatively small, the contacts between them should not be particulartly unfavourable, and could expectedly be compensated by crystal packing.
